# Study on postpartum estrus of guinea pigs (*Cavia cobaya*) using *Anredera cordifolia* leaf extract

**DOI:** 10.14202/vetworld.2017.375-379

**Published:** 2017-04-04

**Authors:** D. Wijayanti, E. T. Setiatin, E. Kurnianto

**Affiliations:** Department of Animal Science, Faculty of Animal and Agricultural Sciences, Diponegoro University, Tembalang Campus, Semarang 50275, Central Java, Indonesia

**Keywords:** *Anredera cordifolia*, *Cavia cobaya*, estrus cycle, postpartum

## Abstract

**Aim:**

The purpose of this study was to determine the postpartum estrus cycle of guinea pigs (*Cavia cobaya*) using *Anredera cordifolia* leaf extract.

**Materials and Methods:**

Materials used were 8 males and 8 females of *C. cobaya* with body weight ranged 400-450 g. Mating ratio applied was 1:1. Treatments given were 0, 10, 50 and 90 mg of *A. cordifolia* leaf extract/head, designated as T0, T1, T2 and T3, respectively. Two females were subjected for each treatment. *A. cordifolia* leaf extract was administered orally from 10 days prepartum to 10 days postpartum. Observation of mating behavior, vulva morphology, and vaginal smear preparation was done in the afternoon for 10 days subsequence postpartum. Data were analyzed by univariate method and descriptively.

**Results:**

The results showed that the addition of *A. cordifolia* leaf extract 50 mg orally could accelerate the time of postpartum estrus based on the average frequency of mating behavior consisting of behavioral approach, allow the buck to sniffing her, mating positions, standing heat, lordosis position, and copulation. During estrus, vulva morphology was red color, had much mucus and no thin membrane covering vagina. There were a lot of superficial cells on vagina.

**Conclusion:**

The best treatment to accelerate occurring postpartum estrus was the addition of *A. cordifolia* leaf extract as many as 50 mg/head weight (T2) orally.

## Introduction

Decreasing livestock productivity was due to the physiological disorder of reproduction postpartum in long time healing of uterus wound. Long time uterus involution could lead to the emergence of long time of postpartum estrus. Farmers usually treat povidone iodine in the postpartum uterine wound. Povidone iodine could damage the monocytes, granulosa, and fibroblast cell tissue [1] because it could pervade on to nets that were not exposed to the wound which was resulting in damage to other tissues [2,3]. Based on that situation, the alternative herbs that were easily available, cheap and non-toxic should be taken. *Anredera cordifolia* was an herb that has benefits in curing various mild or severe diseases, including as a cure wounds [4].

*A. cordifolia* contained saponins, flavonoids, alkaloids, triterpenoids, protein, vitamin C, and phytoestrogens [5-7]. Components of *A. cordifolia* suspected in wound healing uterus were flavonoids, saponins, vitamin C, and phytoestrogens. Flavonoids as antioxidants and anti-inflammatory exogenous that protecting wound from free radicals that could damage cell tissue and inflammatory lesions [8]. Vitamin C as an antibacterial could damage the membrane of bacteria and saponin had a function in the formation of collagen tissue in wound healing [9]. Research conducted by Miladiyah and Prabowo [10] showed that the ethanol extract of *A. cordifolia* leaves with the concentration more than 20% could accelerate the healing of cuts wound in mice compared to povidone iodine.

The faster heal of uterus wound, faster occurring postpartum estrus can be observed from the mating behavior, vulva morphology, and vagina smear. The objective of this study was to observe postpartum estrus of *Cavia cobaya* using *A. cordifolia* leaf extract.

## Materials and Methods

### Ethical approval

The use of material in this study has been approved by Animal Ethics Committee for Using Animal and Scientific Procedures in Faculty of Animal and Agricultural Sciences, Diponegoro University, Indonesia.

### Materials

Materials used were 8 males and 8 females of *C. cobaya* with body weight ranged 400-450 g.

### Methods

#### Experimental procedure

Mating ratio of male:female applied was 1:1. *A. cordifolia* leaf extract dose was a conversion of the human body weight into *C. cobaya* [11]. Treatments given were 0, 10, 50 and 90 mg of *A. cordifolia* leaf extract/head, designated as T0, T1, T2 and T3, respectively. Two females were subjected for each treatment. *A. cordifolia* leaf extract was administered orally from 10 days prepartum to 10 days postpartum.

#### Determination of total flavonoid content

Total flavonoid content was determined by spectrophotometry method by Astuti *et al*. [9]. The content of total flavonoid was expressed in gram per 100 g subfraction (% w/w EK). A total of 500 g of fresh *A. cordifolia* samples leaf was extracted with 5000 ml of 70% ethanol, put into an Erlenmeyer for 5 days with stirring about 15 min, then was filtered to separate the filtrate from the pulp. Then, the filtrate was evaporated to obtain a thick extract. Viscous extract obtained was weighed and stored in the refrigerator before used [5,7]. *A. cordifolia* leaf extract was given orally during 10 days prepartum and during 10 days postpartum.

#### Observation of mating behavior

Mating behavior of *C. cobaya* was observed from 04:00 to 06:30 pm [11,12] for 10 days subsequently postpartum. Parameters observed were behavioral approach, allow the buck to sniffing her, mating positions, standing heat, lordosis position, and copulation [12].

#### Observation of vulva morphology

Vulva morphology of *C. cobaya* was taken during 10 days postpartum. Vulva was seen physically and the vulva picture was taken every day. Each of vulva morphology picture was used to category the vulva in the estrus phase; those were either proestrus, estrus, metestrus, or diestrus phase [13].

#### Preparation of vaginal smear

The vaginal smear preparation was modified method of Leigh *et al*. [14]. Vaginal mucus was taken during 10 days postpartum. Cotton bud was lubricated using physiological saline, then it was inserted to vagina for obtaining vaginal mucus. Vaginal mucus was lubricated in object glass, then it was dried. The fixation of dried preparation was conducted using methanol and then it was dried again. After that, it was colored by using Giemsa staining for 30 min, then it was washed by distilled water and dried. Determination of the estrus cycle vaginal smear was determined estrus cycle phase through the identification of epithelial cell morphology. Epithelial cells were observed parabasal cell shape, intermediate cells and superficial cells as well as the presence of leukocytes different in each phase [15].

### Data analysis

Data were analyzed by univariate method and descriptively for determining the effect of treatment on the observed parameters (mating behavior, vulva morphology, and vaginal smears) [16].

## Results

### Mating behavior

Table 1 shows that mating behavior (frequency and day) consisting of buck approaching, sniffing, standing heat, mating position, lordosis position and copulation showed by T2 were 46 times and day-3, 35 times and day-4, 34 times and day-3, 8 times and day-5 and 4 times, and day-3, respectively. The stand behavior affected by T2 and T3 were in the same frequency, that was 12 times (Table-1) but the occurrence of stand behavior was in the different day (T2 was day-5 and T3 was day-4). Giving *A. cordifolia* during 10 days prepartum affected the return of postpartum estrus phase frequently and fastly occurred at T2 (Table-2). Giving 50 mg *A. cordifolia* leaf extract/head could accelerate uterus wound healing postpartum so that the postpartum estrus was early appear. The readiness of female *C. cobaya* accepting the copulation from male was indicated that uterus was healed.

**Table-1:** The best frequency of mating behavior on *C. cobaya* after giving of *A. cordifolia* leaf extract during 10 days prepartum.

Treatment	Mating behavior											

	Receptive to a buck approaching		Allow the buck to sniffing her		Standing heat		Mating position		Lordosis position		Copulation	
					
	Frequency	Day	Frequency	Day	Frequency	Day	Frequency	Day	Frequency	Day	Frequency	Day
T0	37	5	21	7	10	7	26	9	7	6	3	5
T1	44	4	22	2	10	7	21	2	4	7	2	7
T2	46	3	35	4	12	5	34	3	8	5	4	3
T3	38	7	20	2	12	4	33	5	8	6	1	6

T0=Without giving *A. cordifolia* leaf extract, T1=10 mg of *A. cordifolia* leaf extract/head, T2=50 mg of *A.* *cordifolia* leaf extract/head, T3=90 mg of *A. cordifolia* leaf extract/head. *A. cordifolia*=*Anredera cordifolia*, *C.* *cobaya=Cavia cobaya*

**Table-2:** The best frequency of mating behavior on *C. cobaya* after giving of *A. cordifolia* leaf extract during 10 days postpartum.

Treatment	Mating behavior											

	Receptive to a buck approaching		Allow the buck to sniffing her		Standing heat		mating Position		Lordosis position		Copulation	
					
	Frequency	Day	Frequency	Day	Frequency	Day	Frequency	Day	Frequency	Day	Frequency	Day
T0	28	9	28	8	8	6	24	6	4	2	1	6
T1	34	9	26	7	24	7	26	2	5	3	2	6
T2	38	6	39	7	28	6	36	5	8	5	3	3
T3	35	7	30	8	24	8	33	7	8	3	2	5


T0=Without giving *A. cordifolia* leaf extract, T1=10 mg of *A. cordifolia* leaf extract/head, T2=50 mg of *A. cordifolia* leaf extract/head, T3=90 mg of *A. cordifolia* leaf extract/head. *A. cordifolia=Anredera cordifolia*, *C. cobaya=Cavia cobaya*

Treating with *A. cordifolia* leaf extract containing flavonoids accelerated the appearance of estrus behavior compared to *C. coba*ya without giving extract *A. cordifolia*. Flavonoid would clean the wound by phagocytosis. The next phase was the proliferative phase which would form new tissue and blood vessels.

### Vulva morphology

**Figure-1:**
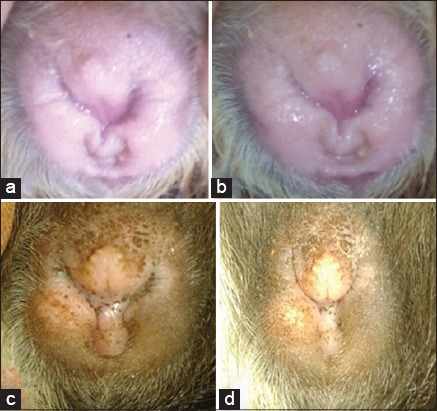
Vulva morphology of *Cavia cobaya* based on estrus phase after giving 50 mg/head orally. (a) Proestrus, (b) estrus, (c) metestrus and (d) diestrus.

Figure-1 showed the morphology of vulva given 0, 10, 50 and 90 mg of *A. cordifolia*. Giving of *A. cordifolia* leaf extract as many as 50 mg/head was the best since it could accelerate postpartum estrus at day-2 compared to day-4 (T0), day-6 (T1), and day-5 (T3). Appearing estrus was fastest by T2 as shown by vulva morphology. *C. cobaya* vagina still had a thin membrane that covered the vagina, vulva color was white, but there was mucus around vulva indicating *C. cobaya* was in proestrus phase (Figure-1a). Vagina covered by a thin vaginal membrane showed that *C. cobaya* was not in estrus. Figure-1b showed there was not thin membrane covering the vaginal hole. Besides, the color of dark red vulva and lots of mucus were available around the vaginal mucus. Estrus was marked by reddish vulva due to blood pressure in the vagina. Figure-1c showed that *C. cobaya* vulva looked reddish pink with longitudinal folds in the edges of the vulva and slightly moist. Diestrus phase (Figure-1d) showed the vulva holes was smaller and palidness.

### Vaginal smear

**Figure-2:**
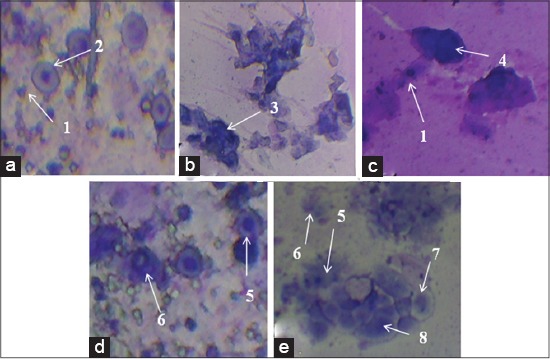
Cell types by vaginal smear in *Cavia cobaya* after giving 50 mg/head orally. (a) Proestrus: (1) Erythrocyte and nucleated superficial cells (2); (b) proestrus-estrus: (3) Enucleated superficial cells; (c) estrus: (1) Erythrocyte and (4) the superficial cells pyknotic nucleus; (d) metestrus: (5) Parabasal cells and (6) neutrophils; (e) diestrus: (5) Parabasal cells, (6) neutrophils, (7) small and medium cells, (8) major intermediate cells.

Vaginal smear observation was conducted to determine the beginning of estrus cycle. The return of postpartum estrus phase by T2 was at the 3^rd^ day of giving *A. cordifolia* extract, in which it showed fastest postpartum (Figure-2). In T0, T3 and T1, appearing postpartum estrus phase was at the day of 5, 6 and 7 days (figures unpublished). This proved that giving of *A. cordifolia* 50 mg extract/head showed antioxidants affect counteract free radicals. If the inflammation wound gradually recovered, then the expectation was the postpartum uterine wounds could heal faster and sooner so that the estrus would sooner appear. At the estrus peak, it would be an increasing of estrogen and decreasing of progesterone hormones followed by the appearance of vaginal epithelial cells.

During proestrus phase (Figure-2a), there were superficial nucleated cells and erythrocytes. Meanwhile, in proestrus-estrus phase (Figure-2b) showed the enucleated superficial cell nucleus. In estrus phase (Figure-2c), there were the dominant superficial cells with pyknotic nucleus in which the erythrocytes existed, but parabasal cells were not exist. Metestrus phase (Figure-2d) showed the neutrophils and parabasal cells in high numbers. Furthermore, diestrus phase (Figure-2e) showed parabasal cells, more neutrophil, major intermediate cells, small cells, and medium. In Figure-2b, enucleated superficial cells developed into pyknotic nucleus superficial cells (Figure-2c).

## Discussion

According to Limaa *et al*. [17], giving a *Glycine max* (L.) Merr could inhibit breast pain and perimeum tenderness in postpartum of women. Sveberg *et al*. [18] stated that the appearance of copulation behavior indicates that animal experience estrus with high estrogen levels. Protein in *A. cordifolia* could stimulates nitric oxide, which increased blood flow carrying nutrients to each cell tissue and it could stimulate the body produce growth hormones and reproductive cells replace the damaged cells [19]. According to Mulaudzi *et al*. [6], flavonoids containing antimicrobial and anti-inflammatory in the wound would interact with the bacteria *Escherichia coli*, *Klebsiella pneumoniae*, and *Staphylococcus aureus* that inhibit bacterial growth. Sukandar *et al*. [20] showed that microbial growth could be inhibited by flavonoids affected the reduction of the damaged tissue so that the healing process could be accelerated. Flavonoids were also as the antioxidants inhibiting free radicals in the form of lipid peroxide so that it prevent the appearance of cell necrosis and increase the vascularization wounds. Inhibition of lipid peroxide was to enhance the vascularization of the collagen fibers and cell damage [21]. Vinothapooshan and Sundar [22] stated that the wound healing process started from the phase of hemostasis by narrowing the blood vessels to stop bleeding in wounds.

Stages fibroblasts by assisting of saponin and vitamin C were to produce collagen which was useful for the formation of new tissue and form fibrin threads [23]. Miladiyah and Prabowo [10] stated that flavonoids and vitamin C have anti-inflammatory properties that could reduce pain in burns. According to Tshikalange *et al*. [24], the compound had the capability against positive and negative gram bacteria which became more susceptible to inhibitory effect and was used in the treatment of sexually transmitted diseases. According to Hubrecht and Kirkwood [25], the estrus cycle of *C. cobaya* ranged 16-19 days and the estrus period was 6-11 h.

*A. cordifolia* leaf extract could increase levels of estrogen causing the appearance of estrus. Flavonoids in *A. cordifolia* also contained phytoestrogens playing a role in stabilizing hormonal function by inhibiting the activity of overload estrogen and could also substitute estrogen in the body when the levels of estrogen were low [9]. Vulva could swell and a thin membrane that covered the vulva disappeared if *C. cobaya* in estrus phase. Feyen *et al*. [26] stated that in estrus conditions; the vulva would looked swollen and received a study. Doraiswami *et al*. [27] stated that *A. cordifolia* leaf extract could improve cervical mucus for the content of flavonoids maintaining the quality and quantity of cervical and vaginal fluid. Isoflavones were part of the flavonoids might be a phytoestrogen compounds, a structure similar to endogenous estrogen, but it gave the mixed effect between estrogenic and anti-estrogenic effects [9]. Phytoestrogens work as an estrogen that might affect the production and breakdown of estrogen by the body, and also the levels of estrogen carried in the bloodstream [17,23,28]. Champlin *et al*. [13] stated that in the metestrus phase, the tissue in the vagina looked pale and dry, in which there were no longitudinal folds around the vulva. Feyen *et al*. [26] stated that low estrogen levels caused females did not want to be approached by male, and the absence of mucus in around of outside of the vulva.

Flavonoid in the *A. cordifolia* leaf extract of 50 mg could accelerate the appearing estrus cycle on *C. cobaya*. According to Hubrecht and Kirkwood [25], the estrus cycle of *C. cobaya* ranged 16-19 days and the estrus period was 6-11 h. Astuti *et al*. [9] stated that flavonoids in *A. cordifolia* as an antioxidant and anti-inflammatory preventing damage blood vessels and chronic inflammation in wound. According to the report of Limaa *et al*. [17], the provision of *G. max* (L.) Merr. in postpartum women as much as 4% (1 g/day) containing isoflavones could ripen vaginal epithel cells from undeveloped superfial cell toward increasing superficial cells. According to Lorenzen *et al*. [29], the morphology of the vaginal smear could be seen in the form of cells parabasal by the small cells form with darker cytoplasmic than the medium cell, the large nucleus and the superficial cells with three different morphologies (nucleation, enucleated, and pyknotic nucleus). It was stated by Bachmann *et al*. [30]; the use of vaginal tablets with a 25 and 10 mcg for women could reduce vaginal pH, improve urogenital atrophy, increasing maturation of vaginal epithelial cells with the discovery of superficial cells in large quantities. According to the report of Limaa *et al*. [17], in hapir pigs, there were no neutrophils before puberty and there was a superficial cells when entering estrus phase and decreased parabasal cells. In mice, the number of neutrophil increased when entering ovulation and there were many superficial cell number in the time of estrus phase while in monkey and humans there were a few number of neutrophils [31].

## Conclusion

On the basis of mating behavior, vulva morphology and vagina smear, it could be concluded that the best treatment to accelerate occurring postpartum estrus was the addition of *A. cordifolia* leaf extract as many as 50 mg/head orally.

## Authors’ Contributions

This work was carried out in collaboration between all authors, DW, EK and ETS: Designed the experimental procedures, DW, EK and ETS: Conducted the research work. ED and ETS: Helped in prepared figure, tables, revised and submitted the manuscript. All authors read and approved the final manuscript.
